# Fine-Grained 3D Modeling and Semantic Mapping of Coral Reefs Using Photogrammetric Computer Vision and Machine Learning †

**DOI:** 10.3390/s23156753

**Published:** 2023-07-28

**Authors:** Jiageng Zhong, Ming Li, Hanqi Zhang, Jiangying Qin

**Affiliations:** 1 State Key Laboratory of Information Engineering in Surveying, Mapping and Remote Sensing, Wuhan University, Luoyu Road No. 129, Wuhan 430079, China; zhongjiageng@whu.edu.cn (J.Z.); hqzhang@whu.edu.cn (H.Z.); jy_qin@whu.edu.cn (J.Q.); 2 Institute of Geodesy and Photogrammetry, ETH Zurich, 8093 Zurich, Switzerland

**Keywords:** underwater photogrammetry, deep learning, semantic segmentation, coral reefs, 3D analysis

## Abstract

Corals play a crucial role as the primary habitat-building organisms within reef ecosystems, forming expansive structures that extend over vast distances, akin to the way tall buildings define a city’s skyline. However, coral reefs are vulnerable to damage and destruction due to their inherent fragility and exposure to various threats, including the impacts of climate change. Similar to successful city management, the utilization of advanced underwater videography, photogrammetric computer vision, and machine learning can facilitate precise 3D modeling and the semantic mapping of coral reefs, aiding in their careful management and conservation to ensure their survival. This study focuses on generating detailed 3D mesh models, digital surface models, and orthomosaics of coral habitats by utilizing underwater coral images and control points. Furthermore, an innovative multi-modal deep neural network is designed to perform the pixel-wise semantic segmentation of orthomosaics, enabling the projection of resulting semantic maps onto a 3D space. Notably, this study achieves a significant milestone by accomplishing semantic fine-grained 3D modeling and rugosity evaluation of coral reefs with millimeter-level accuracy, providing a potent means to understand coral reef variations under climate change with high spatial and temporal resolution.

## 1. Introduction

Coral reefs represent remarkable ecosystems in warm tropical and subtropical oceans, characterized by exceptional biodiversity, structural complexity, and exceptionally high primary productivity [1,2]. These reefs are vital natural resources for humanity and marine ecosystems. However, coral reefs face escalating challenges from global climate change, compounded by localized stressors such as sedimentation, resource extraction, over-fishing, and land-based pollution [3,4]. Alarming statistics reveal that 14% of corals have been lost over the past decade, and, 70–90% of corals could face extinction without effective global warming control measures to limit the increase to within 1.5 °C by 2050 [5,6,7]. Coral reefs face multiple causes of degradation under the aforementioned stresses, necessitating our understanding and minimization of the pressures they endure. Advanced technologies such as photogrammetric computer vision and machine learning are crucial for mapping, monitoring, and modeling coral reefs to enhance our understanding and protection. Furthermore, long-term observations and monitoring are necessary to identify habitats with a high survival potential for conservation. These efforts aim to scientifically assess suitable habitats for sustaining coral reef ecosystems.

The conventional approach of conducting manual local in situ underwater surveys has historically been the primary method for assessing coral distributions and growth health. However, this method requires a significant investment of time during field diver surveys, leading to limitations in the spatial and temporal scales at which ecological surveys can be conducted [8]. Over the past decade, advancements in mapping benthic habitats have been made through satellite and aerial photogrammetry and remote sensing techniques [9,10,11]. These methods offer a rapid means of acquiring information for large-scale coral-monitoring projects and enable the identification of various benthic function types on coral reefs. Nonetheless, due to the water surface effect and pixel mixing, they cannot provide detailed and accurate observations of the complex structures of coral reefs [8,12]. The emergence of underwater photogrammetry and unmanned underwater vehicles have significantly enhanced data collection capabilities for underwater surveys, facilitating the high-resolution monitoring of coral reef observations at millimeter-level precision and enabling the observation of individual corals [13,14,15]. However, it also introduces challenges related to data processing bottlenecks and technical complexity, which are difficult to handle manually using traditional methods [16]. Fortunately, recent rapid progress in the repeatability and accessibility of photogrammetric computer vision and machine learning-based automated tools has gradually alleviated some of these barriers [17]. As a result, this progress holds potential in addressing long-standing monitoring challenges associated with capturing rapid changes in coral reefs with high spatiotemporal resolution and reproducibility. Furthermore, it aids in understanding the vulnerability and resilience of coral reefs in the face of both global and local stressors [18,19].

In photogrammetric computer vision, many approaches have emerged, offering automated image-processing tools to generate high-resolution 3D models that effectively capture the spatial structural complexity of coral reef ecosystems [20,21,22,23]. However, limited by the performance of image processing algorithms and computer capabilities, the changes and trends in coral cover have always been regarded as important biological indicators by the scientific community. Consequently, coral reefs have typically been studied as two-dimensional systems. However, it is evident that the percentage of coral cover is insufficient to reflect the structural complexity of coral reefs. The three-dimensional nature of coral reefs constitutes the diversity of the ecosystem, highlighting the necessity to study structurally complex coral reefs accurately within a three-dimensional space [24]. For evaluating the structural complexity of reefs at small-to-medium (such as millimeter to centimeter) spatial scales, 3D rugosity is a valuable metric closely linked to the high organismic diversity present on coral reefs [18,25]. The Vector Ruggedness Measure (VRM) stands as a widely adopted metric for quantifying surface roughness, integrating the variations in slope and aspect into a unified measurement [26,27]. During the past decade, Simultaneous Localization and Mapping (SLAM) or Structure-from-Motion (SfM) techniques have been utilized to process underwater coral images, enabling the acquisition of fine-grained 3D reconstructions of coral reefs. These techniques have proven valuable in enhancing our comprehension of various aspects, including the spatial clustering of species and the impacts of disturbances on the complexity and community structure of coral reefs [28].

Photogrammetric techniques enable the accurate detailed 3D reconstruction of the structure of coral reefs, but a further intelligent understanding of corals requires the utilization of image-classification techniques. Significant advances in machine learning have greatly improved image segmentation and object classification. Within modern machine learning, recent progress has been achieved in training automated classifiers to classify and segment underwater images for the purpose of quantifying species abundance in marine ecosystems [17,29]. Traditional machine learning methods such as Support Vector Machines (SVMs) and k-nearest neighbors, as well as new deep learning techniques, have been used to estimate coral percent cover, which refers to the percentage of surface occupied in the surveyed area by a given taxa or substrate when viewed from overhead [17]. These classifiers demonstrate promising outcomes for most coral reef classes based on local texture and color features. Notably, the best-performing traditional machine learning classifiers, specifically SVMs, exhibit high accuracy in classifying abundant classes, reaching approximately 80% [30]. However, their accuracy significantly declines when dealing with less common classes, and they are only capable of resolving coral classes at the genus or functional group level. In recent studies, Convolutional Neural Network (CNN)-based classifiers have shown superiority over traditional classifiers in segmenting coral images [31]. Patch-based CNNs are one of the prevalent semantic segmentation methods [32]. However, it is important to note that patch-based CNN models have limitations in terms of classification granularity, as they assign a single class label to an entire patch within an image. To overcome this limitation, the Fully Convolutional Neural Network (FCNN) was introduced, offering modifications to traditional CNNs that enable the full semantic segmentation of input images at the pixel level [32,33,34]. Unlike patch-based CNN, FCNN can provide a classification for each individual pixel within an image. In addition, recently there has been the emergence of general-purpose large models for segmentation, such as Segment Anything (SAM) [35], which have shown good performance in common scenarios. However, their effectiveness is limited when applied to specific scenarios, indicating the need for further research to enhance their performance. Advanced classification techniques have been applied to classify 3D reconstructions of coral reefs, offering valuable insights into the spatial distribution relationships among coral taxa and presenting more realistic representations of organism biomass within coral reef systems compared to two-dimensional metrics such as coral percent cover [17,20]. However, previous studies did not take into account the use of underwater control points in the classification of 3D coral reconstructions, nor did they consider pixel-wise semantic segmentation results to analyze intraspecific and interspecific changes in corals. Therefore, it was impossible to accurately monitor changes in coral reefs with high spatial accuracy over time.

To gain a thorough understanding of the variations and trends in coral reef growth, we employ an underwater videography technique to capture detailed imagery of coral reefs. This underwater visual information serves as the foundation for our proposed approach, which combines the technical advantages of photogrammetric computer vision and machine learning to achieve the fine-grained intelligent understanding of coral reef growth. Initially, we leverage advanced photogrammetric computer vision technology to reconstruct coral reefs in three dimensions. This process yields highly accurate and high-resolution outputs such as underwater digital surface models (DSMs), orthomosaics, dense point clouds, and 3D meshes. These outputs provide a representation that is closer to a digital twin of the coral reef environment. Subsequently, we develop a novel deep neural network specifically designed for the semantic segmentation of coral images. This neural network takes patches of orthomosaics and DSMs as the input and intelligently classifies the different components of coral reefs. By analyzing these segmented outcomes across different time periods, we can obtain a deep understanding of the variations in coral reef growth from multiple perspectives. Photogrammetric computer vision and machine learning integration offer the in-depth exploration of coral reef ecosystems. This approach can effectively support long-term monitoring and contributes to coral reef conservation and management.

## 2. Materials and Methods

**Figure 1:**
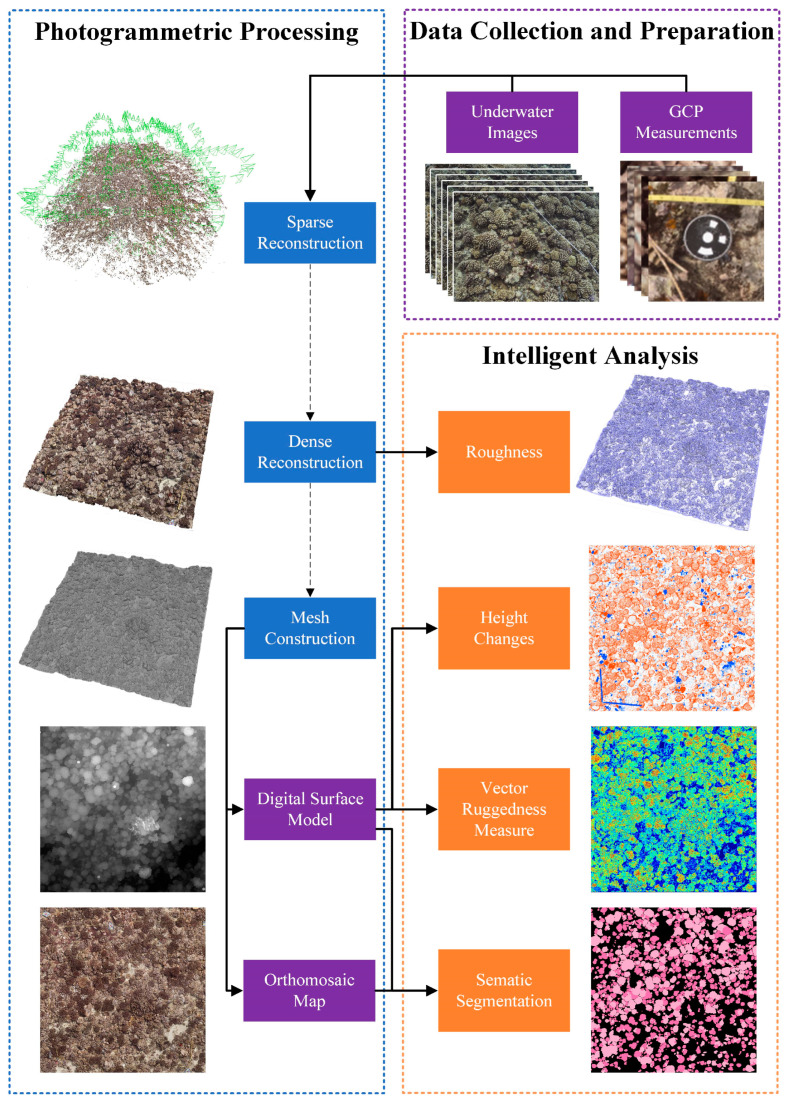
A monitoring workflow for fine-grained coral reef growth.

Our workflow for fine-grained coral reef growth monitoring is illustrated in Figure 1 and comprises three stages: data collection and preparation, photogrammetric processing, and intelligent analysis. The first stage mainly involves the acquisition of underwater images and relevant measurements of Ground Control Points (GCPs). Subsequently, photogrammetric techniques are employed to process the data and generate a set of products, including sparse point clouds, dense point clouds, mesh models, orthomosaics and DSMs. By integrating the semantic segmentation results, intelligent analysis on coral reef growth can be conducted.

### 2.1. Data Collection and Preparation

**Figure 2:**
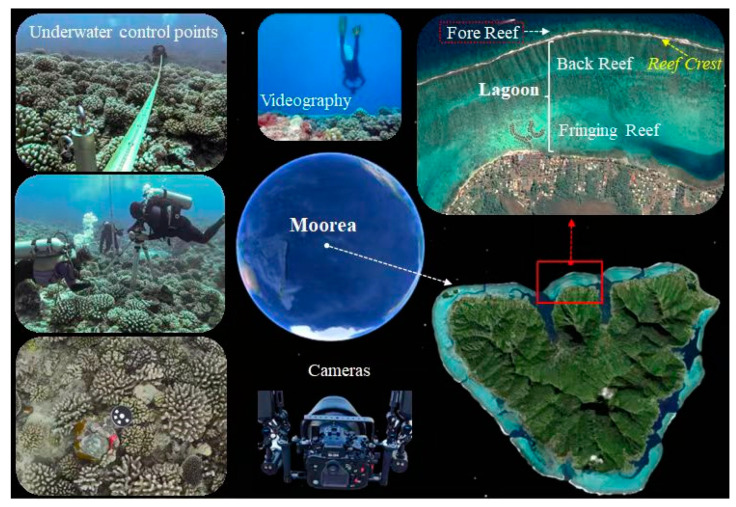
The underwater in situ location and environment.

This study was supported by the Moorea Island Digital Ecosystem Avatar (IDEA) project, which was established in 2013 to create a digital avatar of islands. Moorea island is the core of this project; it is a volcanic oceanic island about the size of San Francisco, with about 10 enclosed coral reefs surrounding the entire island, and there are several of the most complex ecosystems on Earth that can be used to study the impact of natural and anthropogenic stressors on ecosystem processes. Its location is ideal for coral monitoring, where a wide variety of coral observation data can be obtained. In cooperation with the Gump Station, the data used in this study were collected at the fore reef using underwater videography, as shown in Figure 2.

**Figure 3:**
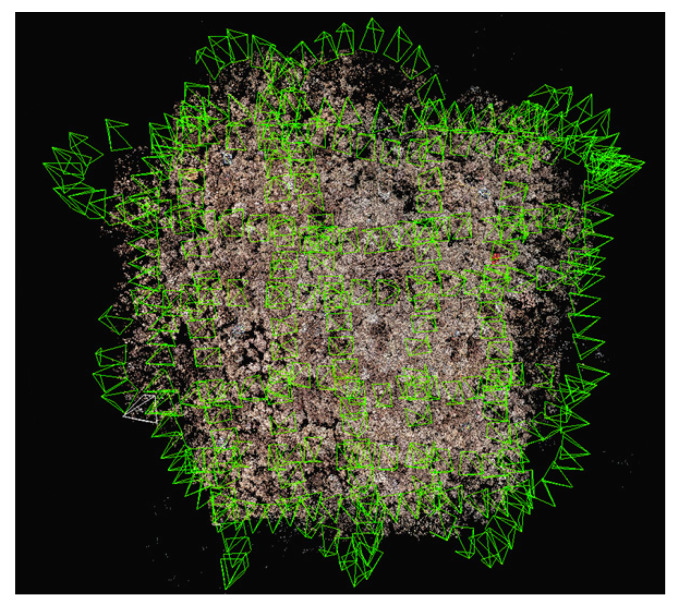
A schematic diagram illustrating the overlapping degree of image acquisition based on the camera’s position and field of view. The green frames in the foreground of the figure represent the position of the camera relative to the object on the coral seabed, while the background consists of the densely colored point cloud of the coral seabed, reconstructed from the coral image.

The device for collecting underwater images is an underwater camera system specially designed for capturing underwater coral images, and its basic information is shown in Table 1. Most underwater images were collected at a height of 2 m from the benthos. To study detailed changes in coral reefs over time, we selected underwater images collected in the same area in August 2018 and August 2019; additional information on underwater images is shown in Table 2. These images were captured along a pre-planned route, and the overlap between adjacent images mostly ranged from 70% to 85%, as shown in Figure 3. As images captured underwater have certain color deviations due to complex lighting, we performed radiometric correction on the acquired images [36]. Specifically, the optical flow procedure introduced by Farneback [37] was firstly applied to estimate the displacements of the red and blue channels with respect to the green channel. Subsequently, the displacements between the channels were used to estimate the parameters of a correction model, namely the collocation model [38]. Finally, the red and blue channels underwent correction, and the color image was re-built.

The dominant coral species that grows in this study area is *Pocillopora*, which is highly sensitive to the impacts of climate change. Consequently, this coral species is considered as an exemplar for conducting coral semantic segmentation and change monitoring analysis. The annotation of training data and ground truth for coral image segmentation on the orthomosaics was performed by expert annotators. Each pixel in the orthomosaics was categorized as live *Pocillopora*, dead *Pocillopora*, and background (comprising other corals, sea rods, algae, stones, sand, etc.). Accurate photogrammetry and monitoring changes in the 3D spatial structure of corals over time were achieved using underwater GCPs on the coral seabed. In the study area, five GCPs are placed, each comprising an aluminum target anchored at a stable position. These targets possess unique patterns that can be automatically recognized and measured using a program. The coordinates of the GCPs are measured using professional measuring instruments. These GCPs establish a single reference datum over all measurement periods and support the self-calibration procedure in bundle adjustment.

### 2.2. Photogrammetric Processing

The photogrammetric products are carried out through an improved photogrammetric computer vision program based on OpenDroneMap, which is a rapidly evolving, community-based open-source photogrammetry project [39]. As illustrated in the left side of Figure 1, its main workflow begins with sparse reconstruction using input images and GCPs to obtain camera poses and a sparse point cloud in the geodetic coordinate system. Dense reconstruction follows to generate a dense point cloud of the scene, followed by mesh construction. Finally, photogrammetric products, including orthomosaics and DSMs, are generated.

**Figure 4:**
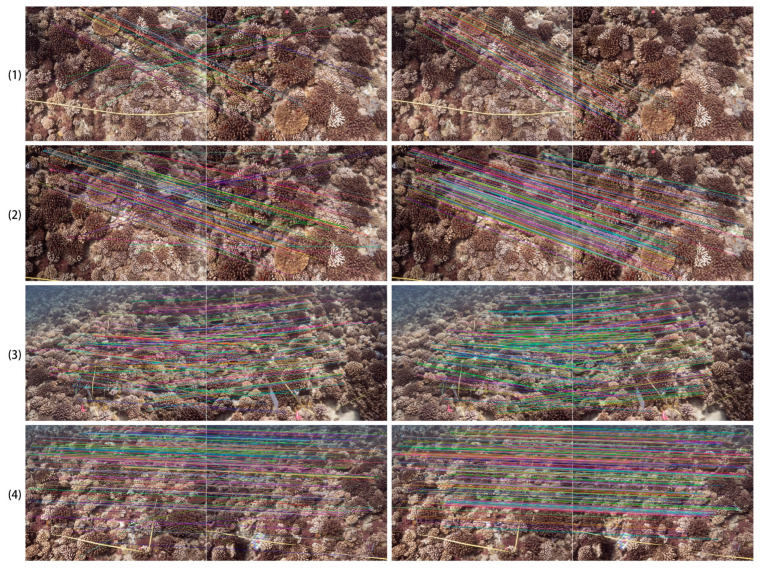
An example for comparison of ratio test (original OpenDroneMap, first column) and AdaLAM (Ours, second column). (**1**–**4**) show the results of four different pairs of overlapped images using these two different feature-matching methods. The colors of the lines are random and used to visually distinguish different matches.

SfM is a core technology for sparse reconstruction in original OpenDroneMap. The accuracy of the SfM reconstruction is crucial for achieving a high-quality final model, which primarily relies on the quality of feature extraction and matching [40]. Unlike common scenes, coral reefs have many complex and similar structures, which can cause repetitive patterns in the captured images and pose challenges for feature matching. If there are many errors in feature matching, it will directly lead to unstable or even failed SfM reconstruction, which highlights the importance of the accuracy and robustness of feature extraction and the matching of underwater coral reef images. In our improved photogrammetric computer vision program based on OpenDroneMap, we conducted feature extraction using RootSIFT [41], an enhanced version of Scale-Invariant Feature Transform (SIFT) [42] that incorporates L1 normalization to enhance descriptor discriminative power, improve matching accuracy under varying illumination conditions, and stands as one of the most widely used and reliable local feature description methods. For feature matching in original OpenDroneMap, the Fast Library for Approximate Nearest Neighbors (FLANN) [43] is commonly utilized. However, FLANN lacks robustness to outliers which can disrupt the nearest neighbor search process, leading to inaccurate matches or affecting the overall quality of feature matching results. In response to this issue, we introduced an advanced adaptive local feature matching method, Adaptive Locally Affine Matching (AdaLAM) [44], which incorporates adaptive thresholds to improve feature matching accuracy and robustness against varying image conditions and geometric transformations. As shown in Figure 4, utilizing AdaLAM for feature matching yields a higher number of correct matches and fewer incorrect matches. Specifically, in our experiments, AdaLAM increases the number of correct matches by approximately 20%, leading to improved stability in SfM reconstruction and higher network coverage of images. The average number of successful feature matches per image and the average number of repeated observations per point are calculated for comparison, as shown in Table 3. When using AdaLAM for feature matching, it is evident that more successful matches can be obtained, while also increasing the number of repeated observations, leading to a more reliable reconstruction.

To enable the quantitative comparison of coral growth in the same location over time, it is crucial to perform geo-referencing that transforms the initial arbitrary datum into a predefined coordinate reference system. As shown in Figure 1, we applied underwater GCPs landmarks and established an underwater geodetic control network using green lasers for leveling and distance measurement equipment (as shown in Figure 2). The geospatial positions (latitude, longitude, and altitude) of GCP landmarks were known. Each GCP can be observed in multiple images, and these observations are used to align 3D models and refine reconstructions, thereby transforming a free-network model into an aligned model [45]. Specifically, five GCPs were deployed in the study area, with four serving as control points for georeferencing and one acting as a check point for accuracy assessment. By calculating the root mean square errors (RMSEs) of check points over the two years, it was determined that the horizontal errors were within 4mm, and the total errors were within 5 mm.

**Figure 5:**
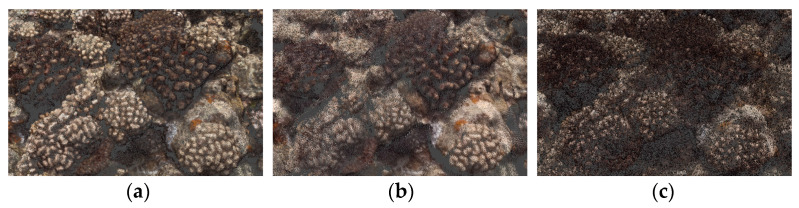
Comparison of dense reconstruction results. (**a**–**c**) display the dense point clouds generated with OpenMVS, Vis-MVSNet and Nerfacto. It can be clearly seen that the point cloud in (**a**) is the most complete, the one in (**b**) is less complete, and (**c**) is the least complete.

After performing sparse reconstruction, the fine structure of the coral reef was constructed through dense reconstruction, and three different reconstruction methods are tested. The first method used the traditional Multi-View Stereo (MVS) algorithm [46] integrated in OpenDroneMap, specifically OpenMVS [47]. The second method adopted a popular deep learning-based MVS algorithm, exemplified by Vis-MVSNet [48]. The third method involved the latest Neural Radiance Fields (NeRF), using the state-of-the-art Nerfacto algorithm [49]. Although NeRF models are not designed to generate point clouds, it is still possible. The visualization results of these three different reconstruction approaches are shown in Figure 5. Overall, the dense point cloud generated by OpenMVS (Figure 5a) is the most accurate and faithful to real-world conditions, capturing rich and precise details. However, there are still some holes caused by occlusions and lighting variations. Vis-MVSNet (Figure 5b) roughly reconstructs the overall shape of the coral reef, but the generated point cloud exhibits non-ignorable errors, rendering the results unreliable. This could be attributed to the use of original pre-trained weights, which might not have included similar coral reef scenes in the training datasets of [48], leading to a subpar performance in this study. The result obtained using Nerfacto (Figure 5c) shows a considerable amount of black noise points, and only a general outline of the coral reef is reconstructed. Since NeRF-based methods perform volumetric rendering based on sampled 3D points, they face the issue of ambiguity in regions with weak local textures due to multiple sampled points of the same color. Furthermore, although NeRF-based methods can achieve similar results to geometric methods in small-scale scenes, they lack effective solutions for large-scale scenes due to limitations such as network capacity, lighting, and image distortions. Currently, NeRF-based methods focus primarily on improving rendering quality, and they still need to be suitable for the high-precision reconstruction of 3D geometric structures in large-scale scenes.

Furthermore, we tested the execution time of the three methods, as presented in Table 4. OpenMVS is implemented in C++, while Vis-MVSNet and Nerfacto are implemented in Python. It can be observed that the traditional MVS algorithm, although yielding good results, requires more computational time. The deep learning-based MVS algorithm demonstrates faster processing speed and has the potential to achieve a comparable or even superior performance to traditional methods in most scenarios in the future. Nerfacto exhibits a fast runtime but produces unsatisfactory results. We attempted to increase the training epochs, but the obtained results were similar. Additionally, there are other high-accuracy reconstruction methods based on NeRF, but they require significantly higher computational resources and have long execution times, resulting in poor practicality.

Upon completion of the dense reconstruction process, dense point clouds and mesh models containing tens of millions of points can be obtained. This further allows for the generation of orthomosaics and DSMs with a resolution of 1 mm. These photogrammetric products possess extremely high resolution and accuracy, providing a solid data foundation for a detailed understanding of coral reef growth variations. Notably, since detailed observation and understanding is the primary consideration in this paper, the Multi-View Stereo (MVS) pipeline is still used for the 3D reconstruction of coral reefs.

### 2.3. Semantic Segmentation

**Figure 6:**
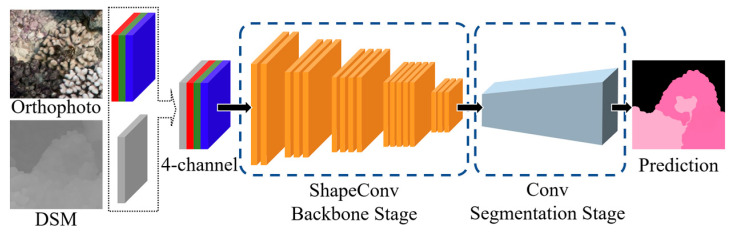
The architecture of MMCS-Net.

Orthomosaic maps of coral reefs have been used as research materials in previous studies on the semantic segmentation of coral reefs [50]. Orthomosaic maps can be considered as high-resolution, detail-rich RGB images that combine actual metric scale, depth information, and geographic coordinates, while correcting distortions in camera imaging. There are many benefits to using orthomosaic maps for semantic segmentation, including reducing the workload of image annotation and more uniform color representation. While conducting semantic segmentation on orthomosaic maps provides a reasonable approach for studying coral species, there are still challenges needing to be solved. Specifically, the complex structure of coral reefs with numerous tentacles results in irregular and complex edges in the images, and the color of corals may be similar to that of the background, such as sand and grass, making high-precision segmentation quite difficult. Moreover, since the images are captured underwater, the changing light caused by wave refraction can also affect the imaging results. Therefore, in these situations, more than simply relying on orthomosaics is required. A practical method is to use the height data provided by DSM, which can complement the color information of RGB images with its geometric information, thereby improving the accuracy and reliability of semantic segmentation. This study proposes a specifically designed network, a Multi-Modal Coral Segmentation Network (MMCS-Net), which takes patches of the orthomosaics and DSMs as the input and generates pixel-level segmentation results. This deep convolutional neural network is capable of handling multi-modal coral observation data to achieve high-precision semantic segmentation. The architecture of our proposed MMCS-Net is shown in Figure 6.

Based on the significant performance achieved by DeepLabv3+ in many common and coral semantic image segmentation tasks and studies, we chose to adopt its architecture as the basis for our network [51,52]. DeepLabv3+ integrates the strengths of an encoder-decoder architecture and atrous spatial pyramid pooling (ASPP), allowing it to capture contextual information from images at different resolutions. Additionally, it incorporates depth-wise separable convolution [53], which reduces parameters and enhances both computational efficiency and classification performance. To leverage the height information from the DSM and improve coral classification performance, we modify the network input from RGB images to RGB images + DSM through channel-wise concatenation. Given the distinct characteristics of the DSM compared to RGB images, the vanilla convolutional layers in the original encoder are replaced by Shape-aware convolutional layers (ShapeConv) [54] to effectively utilize the height information. To integrate RGB and depth cues, ShapeConv incorporates shape information from patches through decomposing the height feature into a shape-component and a base-component, which will cooperate with two learnable weights and be combined by a convolution. In this way, it can help convolutional neural networks achieve performance gains without introducing any computation and memory increase in the inference phase.

In training stage, a hybrid loss function L is applied to obtain high-quality regional segmentation:

(1) $$L=L_{CE}+\mu L_{IoU},$$

where $L_{CE}$ and $L_{IoU}$ denote Cross Entropy (CE) [55] loss and Intersection over Union (IoU) loss, respectively. And μ is a hyperparameter used to balance the weights of different losses. In practice, we discovered that setting μ to 0.4 produces favorable outcomes, so we opted to use this value. The CE loss is one of the most widely used in semantic segmentation. It is defined as a measure of the difference between two probability distributions for a given random variable or set of events, but does not consider the labels of neighborhood and it weights both the foreground and background pixels equally [56]. Unlike cross entropy, which is pixel-wise, IoU is a map-level measure that is originally proposed for measuring the similarity of two sets, and can be used as the training loss [55,57]. Therefore, by integrating these two loss functions operating at different scales, it becomes possible to utilize CE loss to maintain a smooth gradient for all pixels, while using IoU loss to give more focus on the foreground. The implementation of the networks is carried out using the PyTorch deep learning library [58], and the coral segmentation programs are executed on a desktop computer equipped with an NVIDIA GeForce RTX 3090 graphics card and 64GB of RAM.

### 2.4. Geographic Analysis

The photogrammetric processing results include georeferenced 2.5D DSMs and 3D models, with a unified coordinate system and actual metric scale, thus enabling accurate multitemporal geographic analysis. In this study, we conducted a quantitative analysis of coral reef changes between 2018 and 2019, focusing on aspects including height changes, VRM, and 3D roughness. These indicators can reflect coral growth or degradation during these years and offer valuable insights for the study of the long-term changes in coral reef ecosystems. The height changes of the coral reef are directly estimated through pixel-wise subtraction of the DSMs from different years, enabling the creation of the height change map of the reef.

As for surface roughness, it is a critically important measure of reef condition [18], which can reflect the physical structure of the reef and abundance, biomass, and species richness of reef fish. As roughness is a broad concept that can be estimated from various perspectives, we used VRM and 3D roughness to assess the roughness of coral reefs in this study. VRM can be used to investigate spatial patterns derived at high spatial resolution. It is derived from DSM, and incorporates variation of the slope and aspect into a single measurement. In order to calculate the roughness of the terrain, a user-defined floating window is applied to process individual cells. Within each cell, a unit vector orthogonal to the cell is decomposed based on the 3D coordinates of the cell center along with the slope and aspect values. The magnitude of the vector is normalized by dividing it by the number of cells in the neighborhood, and the roughness value is finally scaled with 0 denoting a flat surface and 1 representing the highest degree of ruggedness [16]. In practice, the VRM is computed using the Benthic Terrain Modeler tool (BTM) in ArcGIS (here ArcMap 10.7 is used) [59]. Due to the calculation of VRM within a square window region, its inherent dependence on scale becomes apparent. To comprehensively evaluate the impact of scale in VRM on computational results, the analysis needs to be performed for many different window sizes, from individual polyps to colony scales [27]. As for 3D roughness, it is defined as the distance between a point and the best fitting plane computed on its nearest neighbors. Since 3D roughness is a scale-dependent measure, similar to VRM, it also needs to be computed at multiple scales.

## 3. Experimental Results and Discussion

### 3.1. Analysis and Evaluation of Coral Semantic Segmentation Results

To prepare the dataset, the orthomosaics were partitioned into a series of 448 × 448 patches using a sliding window with a stride of 224. In our study area, nearly 2000 patches can be derived from a single orthomosaic. Subsequently, the dataset was augmented by applying random translations and rotations to enhance its diversity. For the validation procedure, a five-fold cross-validation strategy was applied. The training data were partitioned into five equal subsets (folds), with each fold serving once as the validation set while the remaining folds were used for training and model execution. The model was run five times, and accuracy and loss were computed for each run. In terms of evaluation metrics, the Mean Pixel Accuracy (mPA) and Mean Region Intersection over Union (mIoU) [60] were utilized to report the results. The metrics obtained from multiple tests of each model were averaged to facilitate meaningful comparisons.

**Figure 7:**
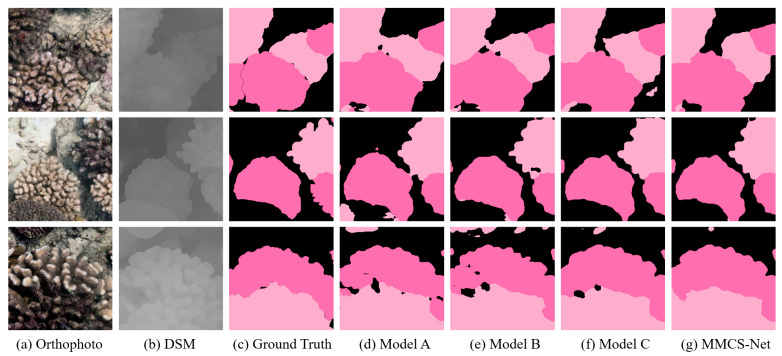
Outputs of different models for a given input: dark pink labels are live *Pocillopora* corals, light pink labels are dead *Pocillopora* corals, and black labels correspond to the background.

To assess the effectiveness of the added DSM and our proposed improvements, we conducted ablation experiments using four different models: (1) Model A, which uses DeepLabv3+ and only takes the orthomosaic patch as input; (2) Model B, which uses DeepLabv3+ and takes both the orthomosaic patch and DSM patch as inputs; (3) Model C, which incorporates DeepLabv3+ but replaces the convolutional layers in the backbone with ShapeConv, utilizing both the orthomosaic patch and DSM patch as inputs; and (4) Our Model MMCS-Net. Models A, B, and C were trained using the Cross-Entropy (CE) loss, while MMCS-Net was supervised by a hybrid loss. The results presented in Table 5 demonstrate that the utilization of DSMs leads to improved segmentation accuracy, and the ShapeConv enhances the processing of DSM data to effectively leverage height information. Our proposed method, MMCS-Net, exhibits the best performance, benefiting from the aforementioned improvements and the hybrid loss. Several semantic segmentation results obtained from the different models are shown in Figure 7. Model A exhibits challenges related to poor lighting conditions caused by occlusions, while Model B demonstrates a slight improvement with the incorporation of height information. By comparing Model B with Model C, it can be concluded that ShapeConv enhances segmentation in edge areas by leveraging structural information more adequately than vanilla convolution. Specifically, ShapeConv facilitates smoother transitions in regions belonging to the same class [54]. MMCS-Net achieves the best overall performance, owing to the integration of ShapeConv and the hybrid loss. Due to the IoU loss in the supervision, MMCS-Net achieves superior overall segmentation results.

**Figure 8:**
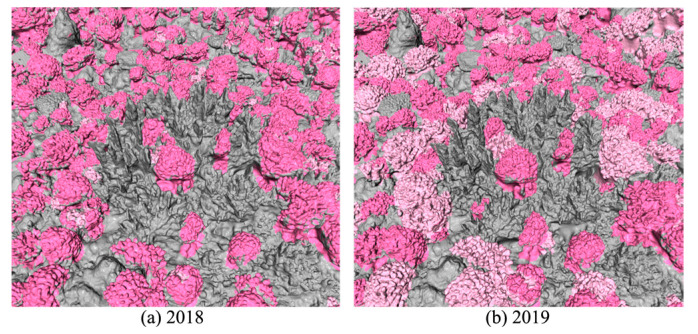
Visualization of the coral mesh models textured with masks from semantic segmentation.

Presenting coral reefs with complex spatial structures in 2D images requires intuitiveness and the ability to show their fine details. Consequently, the exploration of 3D visualization becomes a natural consideration. In this study, we projected segmentation masks obtained from the trained neural model onto corresponding mesh models, as shown in Figure 8. The utilization of these models enables the clear visualization of variations in coral reefs. This approach significantly aids ecologists in studying corals in a three-dimensional, comprehensible, and insightful manner. In Figure 8, live *Pocillopora* corals are represented by dark pink labels, dead *Pocillopora* corals are represented by light pink labels, and the remaining gray labels correspond to the background segments identified through our segmentation process. The observed transition from 2018 to 2019 reveals substantial instances of bleaching or mortality among *Pocillopora* corals. *Pocillopora* corals hold global significance and are particularly susceptible to the ongoing heatwaves in the world’s oceans. The coral data analyzed in this study were collected in August of both 2018 and 2019 using underwater remote sensing technology. Scientists from UC Santa Barbara reported that the heatwave experienced by Moorea Island, which commenced in December 2018 and persisted until May 2019, was one of the strongest marine heatwaves observed in the past three decades [61]. According to their in situ findings, the prolonged heatwave, with temperatures exceeding 29 degrees Celsius, led to the bleaching or mortality of approximately half of the *Pocillopora* corals. This is consistent with the coral variation estimated in our study, highlighting the significance of our approach in efficiently automating the processing of coral observations and performing rigorous quantitative analyses.

### 3.2. New Insights into Coral Height Variation Combined with Semantic Information

**Figure 9:**
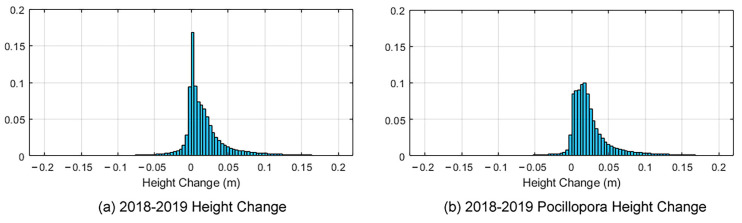
The frequency histogram of our entire study area and *Pocillopora* cover area’s height changes derived from the difference of the DSMs from 2018 and 2019.

Using the generated high-resolution DSMs obtained from different years, it becomes feasible to quantify the vertical changes in coral reef heights. The DSMs from 2018 and 2019 are subtracted firstly, and statistical analysis of the height changes is then performed. Figure 9a depicts the frequency histogram of height changes within the surveyed area. The histogram clearly illustrates a skewed distribution, thereby indicating that the median is a more appropriate measure than the mean for representing the central tendency of the majority of observations [62]. The median of the entire surveyed area was 7.7 mm.

**Figure 10:**
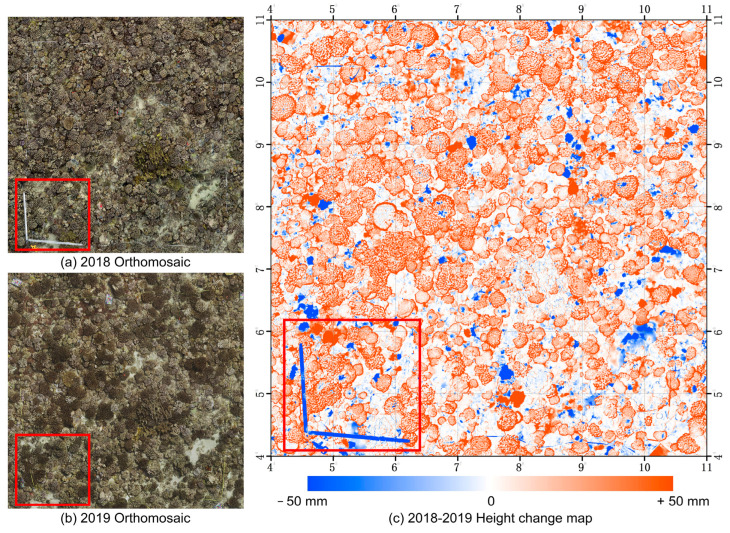
Height change of the coral habitat across the entire study area. (**a**) The orthomosaic map of August 2018. (**b**) The orthomosaic map of August 2019. (**c**) The whole study area height changes measured between August 2018 and August 2019. The size of each grid is 1 m × 1 m.

In order to investigate the spatial distribution of height changes, a height-change map of the entire surveyed area was generated using DSMs from different time periods. As shown in Figure 10c, height changes exceeding an absolute value of 50mm are truncated. The color scheme represents the magnitude of height changes, with red representing an increase, blue representing a decrease and white representing no change. Notably, there is a distinct blue descending area with a near-right-angle shape in the lower left corner of Figure 10c. This region corresponds to manually placed rulers, which were present in 2018 (as shown in the red box in Figure 10a) but not in 2019 (as shown in Figure 10b). The majority of areas are white or very light in color, indicating minimal height change. Only marginal growth is observed at the coral edges, while some corals have even disappeared (as indicated by the blue areas in Figure 10c). These findings strongly suggest that corals in the Moorea Island region are experiencing suboptimal growth under the influence of the heatwave.

**Figure 11:**
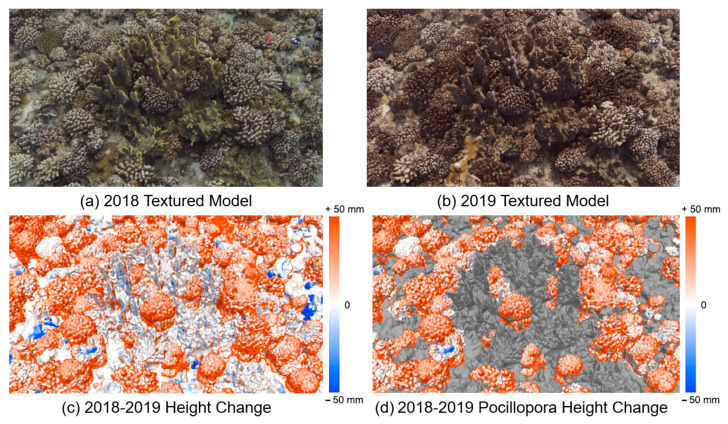
Visualization of coral reef habitat and specific coral species growth changes by integrating semantic information with a 3D model. (**a**,**b**) are the 3D texture model of a piece of the study area in different years. (**c**) Visualization of the whole coral habitat height change mapped to the correspondence 3D mesh. (**d**) The *Pocillopora* coral height change mapped to the same 3D mesh.

By utilizing the semantic mask generated by MMCS-Net, our focus shifted to the height changes specifically related to *Pocillopora* corals. Consequently, a comparative analysis was able to be conducted on the same region of the specific coral reefs in 2018 and 2019. Figure 11a,b presents 3D texture models of a selected piece of coral habitat in the study area in 2018 and 2019, respectively. Figure 11c shows the visualization of the whole coral habitat height change mapped to the corresponding 3D mesh, and Figure 11d highlights the *Pocillopora* coral height change mapped to the same 3D mesh. This approach leverages both the semantic data and the spatial representation provided by mapping to the 3D model to create an informative and visually appealing visualization. It enables detailed representations of geomorphological semantic mapping and facilitates the analysis of spatial variations within the specific coral reef. Moreover, a frequency histogram specifically focusing on the height changes of *Pocillopora* corals was plotted (Figure 9b). The median value increases to 17.8 mm, approximately double the entire study area of 7.7 mm. This finding suggests that although the study area was affected by heatwaves in the latter half of the previous year, leading to widespread bleaching or mortality, *Pocillopora* corals exhibited apparent growth. This observation further elucidates the coral’s ability to thrive and become the dominant coral species within the study area.

### 3.3. Evaluation of Stereoscopic Fine Structure and Complexity of Coral Reef

**Figure 12:**
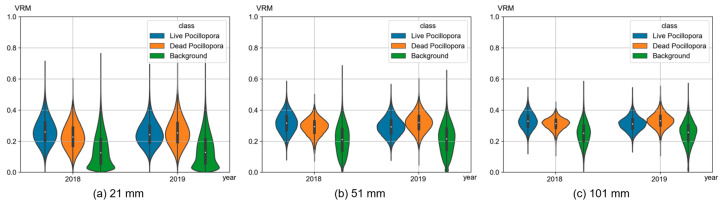
Violin plots of VRMs of different classes (live *Pocillopora* coral/dead *Pocillopora* coral/background) with different window size (21/51/101 mm).

The biodiversity and abundance of benthic fauna are influenced by the fine-scale structural complexity of coral reefs, which can be assessed using VRM and 3D roughness. In this study, the VRM of the coral reef was generated from high-resolution DSMs of 2018 and 2019, with a spatial resolution of one millimeter. Different moving window sizes, representing various resolutions, were applied to investigate the structural complexity across a range of scales, from individual polyps to colony scales. And to study the VRM of different classes, the VRM was calculated for these three classes (live *Pocillopora*, dead *Pocillopora* and background), using the mask generated from coral semantic segmentation. The results were presented in Figure 12 using a violin plot, which allows for the visualization of the distribution of VRM values. It can be observed overall that there are significant differences in the VRM distributions for window sizes of 21 pixels (21 mm), 51 pixels (51 mm), and 101 pixels (101 mm). When the window size is smaller, the VRM is smaller and more dispersed. As the window size gradually increases to 101 mm, the VRM converges towards approximately 0.3. This is because larger window sizes tend to mix coral and background in the calculation, resulting in the convergence of their VRM distributions. At coarser resolutions, the rugosity primarily reflects the slope of the seafloor, whereas finer resolutions reveal a much higher granularity of variation [18]. A lower VRM may indicate the presence of level sand ground, while higher VRM values are indicative of the presence of corals, reefs, and other similar features. It is noteworthy that typical benthic values in natural data tend to be small, typically below 0.4 [59].

Regarding different classes, the VRM of *Pocillopora* is notably higher than that of the background. This is because the set includes sand and aquatic plants, which exhibit lower spatial complexity compared to *Pocillopora*. In 2018, the VRM of live corals was higher than that of dead corals, indicating better growth conditions for live corals over the past year. In contrast, in 2019, the VRM of dead corals showed a noticeable increase compared to the previous year and even surpassed that of live corals. This may be because some larger, faster-growing corals are more susceptible to death from heat waves. Furthermore, they are not covered by algae or other sediments for a short time after death, while live corals are affected by heat waves without significant growth, so there is not much difference in overall spatial complexity between the two of them. That is why VRMs show no obvious differences between a live coral and a dead coral. Usually, within 1 month from bleaching to death, the coral has a white monolithic skeleton with a complete and clear structure; 6 months after death, it will be covered by small algae or thin sediments; 1 to 2 years after death, it will start to corrode. These observations highlight the challenges involved in monitoring rapid changes in coral reefs, requiring the ability to observe and extract information from diverse dimensions and perspectives, alongside the capability to monitor changes with high frequency.

**Figure 13:**
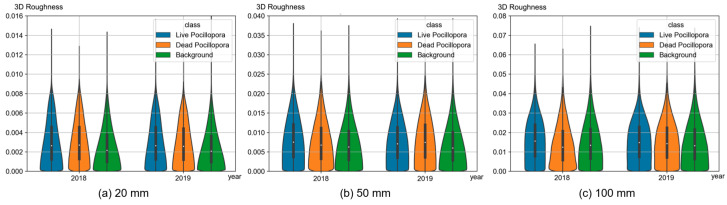
Violin plots of 3D roughness of different classes (live *Pocillopora* coral/dead *Pocillopora* coral/background) with different local neighborhood radii (20/50/100 mm).

**Figure 14:**
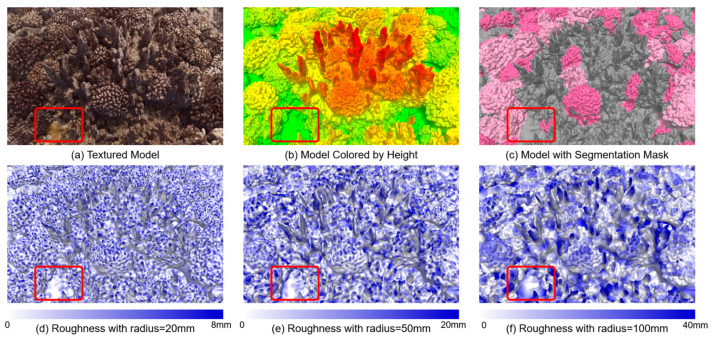
Multi-dimensional stereoscopic visualization results of a typical complex coral reef structure in the study coral habitat. (**a**) The 3D texture model. (**b**) 3D coral model with height color rendering (red indicating high areas and green indicating low depressions). (**c**) The visualization effect of mapping the semantic segmentation results of *Pocillopora* corals to the corresponding 3D habitat mesh model. (**d**–**f**) are the visualization of 3D roughness calculated with different local neighborhood radii.

As for 3D roughness, we also applied different local neighborhood radii (20 mm, 50 mm, 100 mm) during calculation, as illustrated in Figure 13. Unlike VRM, which is derived from DSM data, 3D roughness is computed based on the 3D dense point cloud, so it can reflect the real situation of the 3D structure of the scene more reasonably and is more suitable for a scene with fine and complex structures like coral reefs. The 3D roughness from different neighborhood radii reveals that as the radius increases, the values of 3D roughness demonstrate a nearly proportional increase, particularly for *Pocillopora* corals. When the radius is small, there is a noticeable difference between the 3D roughness of the background and the 3D roughness of *Pocillopora* corals. However, as the radius increases to 100mm, the overall distributions become very similar. This is because when the radius reaches the decimeter scale, it is likely that a significant number of points of corals and background are simultaneously included in the 3D roughness calculation, resulting in a smaller difference between them. The 3D roughness of a specific *Pocillopora* coral coverage area is visualized in Figure 14, revealing distinct patterns. When the radius is 20 mm, the corals exhibit higher roughness primarily at their tentacles, while the rock structures show higher roughness at their edges. As the radius increases, the prominently protruding areas exhibit higher roughness, and the inclusion of points from other regions in the calculation also leads to increased roughness in flat areas. For instance, in the bottom-left corner of the region, there is a flat sandy area. When the radius is set to 20 mm, this area appears white in Figure 14d, indicating low roughness, which aligns with the actual situation. However, when the radius is increased to 100 mm, the surrounding area shows significantly higher roughness compared to the central area (Figure 14f). Therefore, it can be concluded that a centimeter-scale radius is more suitable for investigating the structural characteristics of corals in this particular area. Furthermore, in 2018, the overall 3D roughness of dead *Pocillopora* corals was lower than that of live *Pocillopora* corals, even falling below the background when the radius was 100mm. This is attributed to the limited presence of small *Pocillopora* dead corals in 2018, while the background included some rugged reef structures. In 2019, the 3D roughness of dead *Pocillopora* corals was similar to or slightly higher than that of live *Pocillopora* corals, which closely aligns with the results obtained from VRM analysis.

## 4. Conclusions

This study introduced a novel method that combines photogrammetric computer vision and semantic segmentation techniques to enhance the comprehensive understanding of variations in coral reef growth. The proposed approach was applied to process high-resolution coral images collected through underwater remote sensing in Moorea in 2018 and 2019. It can generate high-resolution orthomosaics, DSMs, dense cloud points, and mesh models of the coral reef, providing a detailed geometric representation of the reef structure. The process of coral semantic segmentation is conducted using a proposed new multi-modal deep neural network that effectively integrates color information from the orthomosaics and structural information from the DSMs. Building upon this foundation, a multi-dimensional intelligent analysis and understanding of coral reef growth can be conducted from both 2D and 3D perspectives. By transitioning from the commonly used 2D metrics to the 3D metrics enabled by our novel method, the more realistic representations of coral reefs can be achieved. For instance, a vertically oriented coral reef may contribute minimally to the percentage cover metric, but may possess substantial biomass that is relevant to metabolic processes, food webs, and other ecological processes. The findings of this study illustrate that the coral reefs located in Moorea Island have undergone detrimental effects such as heatwaves, inadequate growth, and extensive instances of bleaching or mortality. This is consistent with the in situ observed detrimental effects of persistent heatwaves on corals. The utilization of underwater remote sensing data and the extraction of multi-dimensional information will assist coral biologists in further analyzing coral growth and recovery patterns following their exposure to stressors, as well as in identifying coral reef refuges. By adopting this innovative approach, the routine mapping of rapid changes in coral reefs will become more feasible in the near future. The ability to discover and monitor the health, growth, and refugia of coral reefs presents a significant pathway for implementing meaningful conservation interventions, thereby contributing to the protection and preservation of these vulnerable ecosystems in the face of extreme climate change challenges.

## Figures and Tables

**Table 1:** Basic information of the camera system.

Property	Detail
System name	PL51
Camera body	PANASONIC LUMIX GH5S
Sensor type [dimensions(mm)]	Four thirds [17.3 × 13]
Pixel size (μm)	4.6
Lens	Lumix G 14 mm f/2.5
Underwater pressure housing	Nauticam NA-GH5
Port lens	Nauticam N85 3.5″ wide angle dome-port
Resolution	3680 × 2760 pixels

**Table 2:** Additional information of acquired underwater coral images.

Property	Detail	
Year	2018	2019
Specific Date	22 August	21 August
Height from the benthos	about 2 m	
Imagery System	PL51	

**Table 3:** Sparse reconstruction results using different feature matching methods. *Matched Features* refers to the average number of successful feature matches per image, and *Repeated Observations* refers to the average number of repeated observations per point.

Year	2018		2019	
Feature Matching	Default	AdaLAM	Default	AdaLAM
*Matched Features*	8497	9614	7641	8808
*Repeated Observations*	2.99	3.33	2.97	3.24

**Table 4:** Mean execution times.

Method	Time (Minute)
OpenMVS	129.4
Vis-MVSNet	65.1
Nerfacto	10.6

**Table 5:** Performance comparison of different segmentation models.

Setting	mPA	mIoU
Model A (DeepLabv3+)	89.9%	80.5%
Model B	90.8%	82.1%
Model C	91.6%	83.5%
**MMCS-Net (Ours)**	**92.2%**	**84.7%**

## Data Availability

Not applicable.
